# The interplay between stress and physical activity in the prevention and treatment of cardiovascular disease

**DOI:** 10.3389/fphys.2013.00346

**Published:** 2013-11-27

**Authors:** Matthew A. Stults-Kolehmainen

**Affiliations:** Department of Psychiatry, Yale Stress Center, Yale University Medical SchoolNew Haven, CT, USA

**Keywords:** exercise, distress, mental health, physical activity, stress, psychological, cardiovascular diseases

Cardiovascular disease (CVD) continues to menace developed and developing nations alike. However, in the United States alone, 40.5% of the population is projected to have CVD or a closely related disorder by 2030 (Heidenreich et al., 2011). Within the same time frame, costs associated with CVD are expected to balloon up to $818 billion (Heidenreich et al., 2011). These societal strains are increasing parallel to rising mental health disorders and psychosocial stress (Centers for Disease Control, 2011). In this context, stress refers to both physical and emotional challenges, some of which may be transient and rather innocuous (e.g., final examinations, strenuous exercise) with potential positive adaptations. Other stress may be chronic and uncontrollable (e.g., caregiving for a loved one with a terminal illness), which may produce excessive wear and tear, resulting in lasting and harmful insults to one's physical and mental health (McEwen, 2007). The American Psychological Association (APA) reports that 72% of Americans perceived that their stress increased or held steady from 2007 to 2011. Fourth fifths of this population reported an increase from 2010 to 2011 (American Psychological Association, 2012).

Unsurprisingly, there are well-established connections between stress and CVD. Indeed, stress provides an independent contribution to CVD outcomes (Vitaliano et al., 2002). Stress is linked to the pathogenesis of coronary heart disease (CHD) (Rozanski et al., 1999), incidence of acute myocardial infarctions (Rosengren et al., 2004) and worse survival from cardiac events (Kivimaki et al., 2002; Milani and Lavie, 2009). In one eye opening investigation, Milani and Lavie (2009) found that patients with high psychosocial stress in cardiac rehabilitation were almost 4 times as likely to die as those with low stress (22 vs. 5%). The experience of distress, the emotional outcome of stress, also exacerbates the morbidity associated with CVD (Centers for Disease Control, 2011). Those who are objectively stressed, such as caretakers of those with chronic conditions, have higher resting heart rates, blood pressure, and greater incidence of metabolic syndrome (Vitaliano et al., 2002). Individuals reporting higher levels of stressful life events have higher scores on risk factors for CVD, such as smoking (Ansell et al., 2012), systemic inflammation (Puustinen et al., 2011) and obesity (Sinha and Jastreboff, 2013). They are nearly twice as likely to start using anti-hypertensive medication over time compared to less stressed individuals (Rod et al., 2009). Furthermore, those with CVD are often stressed by their condition—adjusting to a life with a long-term hardship (Bodenheimer et al., 2002). This demonstrates that stress both contributes toward disease and, reciprocally, emanates from the experience of disease, which leaves little doubt that mental health interventions are needed throughout the progression and treatment of CVD.

Given these findings, an effective prevention and/or treatment plan for CVD would also target stress, but would minimize additional side effects and healthcare costs. To this end, exercise interventions have proved effective. In terms of CVD pathology, exercise improves the odds of recuperating from stroke, reduces hypertension and diminishes symptoms of heart failure and CHD—in each case with few serious side effects (Kujala, 2009). Recent evidence finds that exercise is just as effective as or even more effective than medications. For instance, Naci and Ioannidis (2013) recently found that exercise had a stronger effect than anticoagulants and antiplatelets in the treatment of stroke. Moreover, there is convincing evidence that those who exercise are much less likely to develop CVD over time (Lee et al., 2012; Matheson et al., 2013). The same is true for depression and post-traumatic stress disorder (PTSD) (Leardmann et al., 2011). In a large military cohort, those who exercised at least 20 min of vigorous activity two times a week were >40% less likely to develop new onset PTSD over >3 year period (Leardmann et al., 2011). Among those experiencing difficulties with stress, aerobic and resistance exercise has been effective in reducing stress-induced cardiac reactivity (King et al., 2002; Faulk and Bartholomew, 2012) and perceived stress (Wilcox et al., 2008), particularly when paired with healthy dietary changes (Imayama et al., 2011) and behavioral modification programming (Atlantis et al., 2004). Given the strong association between stress and CVD, is it possible that part of the health-enhancing power of exercise stems from its ability to mitigate the effects of stress (Milani and Lavie, 2009)? Extensive data supports the thought that physical activity (PA) buffers the relationship between stress and physical health problems (Gerber and Pühse, 2009; Emeny et al., 2012; Hamer, 2012; Rueggeberg et al., 2012). In other words, at high levels of stress, greater PA is associated with better health (Rueggeberg et al., 2012). Additionally, those who are physically fit are more resilient to the effects of stress, such as high work demands, resulting in less heart disease and associated mortality (Holtermann et al., 2010).

Data from our own laboratory show that higher levels of moderate to vigorous exercise are associated with fewer complaints of cardiovascular problems—particularly when stress is low (Stults-Kolehmainen et al., accepted). Under high levels of stress, however, higher levels of exercise were not related to better health, contradicting previous research (Rueggeberg et al., 2012). While PA—and more specifically, exercise—certainly have a salubrious effect on both psychosocial stress and CVD, and lifestyle interventions increasingly emphasize all forms of PA, I was left to wonder about the possible limitations of these health behaviors. One possible explanation for these discrepancies is that various forms of PA may have differential influence on the stress and CVD relationship (Fredman et al., 2006). PA, by definition, is any movement that results in energy expenditure, including occupational and spontaneous forms of locomotion (Garber et al., 2011). This is important because stressed populations, particularly laborers, may engage in moderate to high levels of occupational activity. Exercise, however, is typically performed with the intent of increasing physical fitness. Aside from athletes, military personnel and physical education students, it is usually considered recreational and is completed during one's leisure time. Therefore, it is possible that quantifications of exercise simply capture the luxury of having more time for rest and relaxation (Iwasaki et al., 2001). A preponderance of evidence would support the observations that those with less leisure are more frequently stressed, but also that those who are stressed typically have less leisure time (Fredman et al., 2006; Lutz et al., 2007).

Consequently, in the pursuit of better health, stress may have the upper hand over one's ability to engage in healthful levels of PA (Salmon, 2001). In fact, Rafer Lutz established that stress has a stronger effect on PA than the reverse order of influence (Lutz et al., 2007). This was the first and only prospective design with the specific aim of untangling these effects. Lutz, John Bartholomew and I later published data that suggested that those who are in earlier stages-of-change for exercise (i.e., pre-contemplators, contemplators) are most vulnerable to this effect (Lutz et al., 2010). This association does not appear to be limited to non-habituated exercisers, however. In our recent systematic review, Rajita Sinha and I found that >85% of prospective studies examining the association of stress and PA/exercise reported an inverse relationship between these two constructs. In other words, the experience of high mental stress predicted less PA. Several studies used sophisticated designs to objectively capture periods of greater stress and examine future levels of PA or exercise (Griffin et al., 1993; Steptoe et al., 1996; Vitaliano et al., 1998; Roemmich et al., 2003; Oaten and Cheng, 2005; Smith et al., 2005; Sherman et al., 2009). However, only one investigation employed a true experimental design and manipulated an acute mental stressor (Roemmich et al., 2003). In this study, children participated in two conditions, the order of which was randomly selected. In the stress condition, these subjects were required to give a prepared talk on a social topic. This task induced a moderate to strong stress response, which was followed by the opportunity to be either physically active (riding a bicycle) or remain sedentary. Behavioral responses were compared to changes observed within a passive reading control condition. While responses subjective stress were not related to changes in exercise (*r* = −0.19), children were less likely to expend energy after the stress condition compared to the control (a 21% difference in total minutes of activity). Total energy expenditure was also lower in the stress condition. Also examining this outcome, Smith et al. (2005) found approximately a 1000 kcal difference of weekly activity in a cohort of parents with a child who recently received a cancer diagnosis vs. parents of a healthy control child. Effects sizes for this association were large: 1.71 (Cohen's d) at diagnosis and 1.13 at a 3-month follow up. Such differences were due to less PA and more sedentary behavior, such as TV viewing. Given the long waits in doctors' offices to which these parents are subject, this is not surprising. In another longitudinal case-control study, caregivers had more stress and less frequent exercise bouts over two time points compared to matched controls (Vitaliano et al., 1998).

Following students before and during a final examinations period is a highly convenient method to observe potential influences of stress on health behaviors (Griffin et al., 1993; Steptoe et al., 1996; Oaten and Cheng, 2005; Sherman et al., 2009). In two cases, control groups have been utilized to determine whether other factors could explain changes in PA, such as the weather (Steptoe et al., 1996; Oaten and Cheng, 2005). These studies have identified significant declines in PA compared to controls during examinations, and such changes are due to less frequency, duration, and perceived ease of exercise. In an interesting permutation on this design, Sherman (Sherman et al., 2009) found that PA declined across a 14-day period that terminated with students' most stressful examination—a medium effect (*p* = 0.03, *d* = 0.62; η^2^ = 0.26). Unfortunately, none of these studies looked at changes in PA after the completion of a stressful examination period or over multiple cycles of examinations, which would provide an additional insight on the dynamic effects of stress on exercise behavior. A rebound in PA after a stressful frame of time would complement existing data. Nevertheless, when viewed collectively, the published data substantiate the notion that both acute and transient or chronic life stressors have an impact on facets of PA.

These findings have direct implications for clinicians interested in improving CVD outcomes. Exercise is effective for treating both CVD and stress, but the patient would benefit from a treatment plan that goes beyond a prescription of exercise and “usual care.” Navigating the complexities of a more active lifestyle is stressful in itself, particularly for the uninitiated (Stults-Kolehmainen and Sinha, 2013). Second, those who are chronically stressed experience both poor recovery from exercise and derive less affective benefits, placing them at risk for dropout early after initiation of an exercise routine (Stults-Kolehmainen and Bartholomew, 2012). Exercise cannot help those who do not adhere to a structured regimen, and stressed populations are at risk for drop out and poor compliance with their doctor's prescriptions. For instance, depressed cardiac rehabilitation patients are less likely to adhere to an exercise program; coming to fewer sessions and dropping out at high rates (Glazer et al., 2002). As a metaphor, the sun-scorched plant benefits from water, but inordinate heat will also evaporate the new moisture. Only when shade is introduced can full restoration occur. In a similar light, the human organism needs relief from stress to effectively take advantage of exercise. The combination of PA and stress management would likely have a synergistic effect. In a randomized controlled trial, Blumenthal et al. (2005) demonstrated that exercise training and stress management groups significantly improved flow-mediated arterial dilation and other clinical outcomes compared to a third group receiving only usual care. They did not test the utility of both exercise and stress management combined, which has been successfully tested by Ornish et al. (1998). Their lifestyle intervention resulted in 50% reductions in the re-occurrence of cardiac events. Given this progression, a logical follow-up study should fully intertwine stress management procedures, such as mindfulness-based stress reduction (MBSR) (Ludwig and Kabat-Zinn, 2008), as part of the exercise regimen. Mindfulness practices can be conducted during movement (i.e., controlled breathing and contractions with proper alignment), during rest periods (e.g., focusing on changing internal states) and directly following a workout (e.g., sensing stillness and the process of unwinding). Such a combination may enhance physical recovery (Stults-Kolehmainen and Bartholomew, 2012), boost affective valence (increase positive emotions), and reinforce exercise behavior resulting in more effective treatment with time and cost savings.

Stress is a common denominator underlying CVD and the lack of PA. It independently contributes to pathologies of the cardiovascular system, exacerbates distress associated with these problems and impedes one's progress toward a healthier lifestyle. As this is a complicated set of relationships, I would argue that novel therapeutic regimens are needed to address the manifestation of stress at multiple levels. Fully melding exercise interventions with proven stress management techniques, such as MBSR, would be a useful strategy.
